# Liver-on-a-Chip‒Magnetic Nanoparticle Bound Synthetic Metalloporphyrin-Catalyzed Biomimetic Oxidation of a Drug in a Magnechip Reactor

**DOI:** 10.3390/mi10100668

**Published:** 2019-10-01

**Authors:** Balázs Decsi, Réka Krammer, Kristóf Hegedűs, Ferenc Ender, Benjámin Gyarmati, András Szilágyi, Róbert Tőtős, Gabriel Katona, Csaba Paizs, György T. Balogh, László Poppe, Diána Balogh-Weiser

**Affiliations:** 1 Department of Organic Chemistry and Technology, Budapest University of Technology and Economics, 1111 Budapest, Műegyetem rkp. 3, Hungary; decsi.balazs@mail.bme.hu (B.D.); krareka@gmail.com (R.K.); 2 SpinSplit Llc., 1082 Budapest, Leonardo da Vinci u. 43b, Hungary; k.hegedus@spinsplit.com (K.H.); ender@eet.bme.hu (F.E.); 3 Department of Electron Devices, Budapest University of Technology and Economics, 1117 Budapest, Magyar tudósok krt. 2, Hungary; 4 Department of Physical Chemistry and Materials Science, Budapest University of Technology and Economics, 1111 Budapest, Műegyetem rkp. 3, Hungary; bgyarmati@mail.bme.hu (B.G.); aszilagyi@mail.bme.hu (A.S.); 5 Biocatalysis and Biotransformation Research Centre, Faculty of Chemistry and Chemical Engineering, Babeș-Bolyai University of Cluj-Napoca, 400028 Cluj-Napoca, Arany Janos street 11, Romania; totos.robert@yahoo.com (R.T.); gabik@chem.ubbcluj.ro (G.K.); paizs@chem.ubbcluj.ro (C.P.); 6 Department of Chemical and Environmental Process Engineering, Budapest University of Technology and Economics, 1111 Budapest, Budafoki út 8, Hungary; gytbalogh@mail.bme.hu; 7 Department of Pharmacodynamics and Biopharmacy, University of Szeged, 6720 Szeged, Eötvös u. 6, Hungary; 8 SynBiocat Llc., 1172 Budapest, Szilasliget u 3, Hungary

**Keywords:** drug metabolism, biomimetic oxidation, microfluidics, organ-on-a-chip, liver-on-a-chip

## Abstract

Biomimetic oxidation of drugs catalyzed by metalloporphyrins can be a novel and promising way for the effective and sustainable synthesis of drug metabolites. The immobilization of 5,10,15,20-tetrakis(2,3,4,5,6-pentafluorophenyl)iron(II) porphyrin (FeTPFP) and 5,10,15,20-tetrakis-(4-sulfonatophenyl)iron(II) porphyrin (FeTSPP) via stable covalent or rapid ionic binding on aminopropyl-functionalized magnetic nanoparticles (MNPs-NH_2_) were developed. These immobilized catalysts could be efficiently applied for the synthesis of new pharmaceutically active derivatives and liver related phase I oxidative major metabolite of an antiarrhythmic drug, amiodarone integrated in a continuous-flow magnetic chip reactor (Magnechip).

## 1. Introduction

Continuous flow chemistry is one of the fastest evolving discipline. Its application is widespread in the field of chemistry. It is used not only in petrol chemistry, but also in the commodity chemical industry. Moreover, the interest of the pharmaceutical industry in continuous flow chemistry is getting intensified, because this methodology offers robust and well controlled strategy to produce active substance and drug form [1]. The expenses and time of both, drug development and manufacturing can be decreased with its application offering the easiest way to implement design space approach of drug manufacturing [2,3]. A flow chemistry set is consisted of several components. In the simplest instance, the solutions of reagents are moved through a reactor by a pump. The reactor could contain solid or immobilized catalyst and the flowed-out reaction mass from the reactor is processed, even in other continuous down-stream steps. Continuous flow catalysis opens up the possibility to perform reactions that cannot be performed in traditional, batch circumstances which involves dangerous reagents, unstable intermediates or has extremely high reaction enthalpy [4,5]. The good controllability is due to the great surface to volume ratio, that enables large heat transfer, moreover the plug like operation ensures perfect mixing of the fluid intakes even in laminar conditions, while there is a small amount of liquid in the reactor at the same time. The productivity of tubular reactors differs from the traditional batch reactors. In a continuous reactor the reaction time can be assimilated with the average contact time of the reactants with the catalyst and is specified by the cross-section area and the length of the reactor, by the volume of the catalyst and by the flow rate of the reactants [6]. A disadvantage is that every reagent must be held in solution, no precipitation is allowed because it could cause blocking in the tubing. Continuous flow systems can be classified by the diameter of the reactor. While tubular reactors with over than 500 µm diameter are known as mesoreactors, microfluidics engages microreactors with less than 500 µm diameter [7]. Since the 1970s and the 1980s several microfluidic appliances were appeared such as microfluidic sensors, pumps, and valves characterized this period. The discipline had been evolved since the defining work of Manz and co-workers at the Fifth International Conference on Solid-State Sensors and Actuators (Transducers ’89) [8] where they summarized the possibilities of applications of microfluidics.

It should be noted, that the aim of microfluidics is not to miniaturize the size of the appliances (which can have also benefits), but mostly to reduce the diameter of the flow space, with major impact upon the nature of transport and transfer phenomena. The reason of these observations is the microscopic amount of liquid in the tubing. The “micro” prefix is not meaning the size of the chip or the diameter of the conduits, but the small overall volume that causes the changes in the flow ratio [9].

Microfluidic instruments can be used in a wide range of fields, starting with analytical [10], biological [11], and synthetic usage [12]. Special representations of microfluidic appliances are “Lab-on-a-chip” reactors, which conceptions were established by Burns et al. [13]. Their goal was to create a miniaturized device which contains all the necessary components (pumps, valves, tubing, mixers, sensors, detectors etc.) that is suitable to analyze very small volume (nanoliters) of DNA sample. Hereby they have created an integrated, effective, reliable, inexpensive and compact tool, that can analyze DNA in a short time and can be used widespread not only in medical diagnoses but also in agriculture.

“Organ-on-a-chip” reactors, being a subtype of Lab-on-a-chip family, mimic the (coordinated) operation of the living organism’s one (or more) organ(s). Enzyme catalyzed biotransformations can be run with the use of them in a well-controlled way. With the immobilization of enzymes on solid supports, a heterogeneous catalytic system can be obtained which is stable enough to reuse it for several time [14]. Organ-on-a-chip reactors can be also used in drug and in preclinical drug research, which is traditionally expensive and time consuming. During the research of in vitro cell based and in vivo experiments were carried out to characterize structure-effect relationship. However, the prior methodology is not able to reveal interactions between tissues and cells, whilst the analysis of the latter is difficult and rises ethical issues. With the use of organ-on-a-chip reactors one could ensure a physiologically relevant in vitro system mimicking the in vivo metabolism of drug candidates [15].

In the human body the main metabolic pathway of drugs and xenobiotics are enzymatic biotransformations, which are generally started by oxidation catalyzed by the CYP450 isoenzymes related oxidative metabolism. The CYP enzymes are mixed type monooxygenase enzymes, located in the endoplasmic reticulum of cells, and in high concentration in the liver. The in vitro methods that are used in preclinical studies are based mostly on hepatocytes and liver microsomes. The latter method is used the most often to study the metabolism of drug molecules of the CYP enzymes. However, in these experiments a complex biological matrix is formed due to the necessity of several coenzymes and their regeneration. This complexity of the matrix makes the analysis difficult and allows only quantitative analysis [16,17]. Therefore, such metabolism mimicking in vitro methods are in the focus of research that can simulate the metabolism of drug molecules without the necessity of complex biological matrix, and can produce metabolites directly from the parent molecule. Synthetic metalloporphyrins can be applied for these purposes. Application of metalloporphyrins is based on their structural resemblance to the heme prosthetic group being present within the active site of the CYP enzymes [18,19]. However, the major disadvantage of the synthetic metalloporphyrins is their easy degradation (autooxidation) under homogeneous oxidative conditions resulting in a short lifetime. This situation can be improved by immobilizing them on solid support by covalent binding or by secondary interactions like ionic bond [20,21]. In our recent work, we showed that meso-tetra(parasulphonato)iron porphyrin could be immobilized on aminopropyl group-modified silica by ionic bond, and the bonded catalyst could be applied in packed bed reactor to perform biomimetic oxidations under continuous flow conditions providing metabolites of antiarrhythmic drug, amiodarone [22]. 

Another possibility is to use magnetic nanoparticles (MNPs) as solid support to immobilize metalloporphyrins. MNPs proved to be suitable carrier of several types of catalysts [23,24,25]. Their application opens up the possibility of creating microfluidic magnetic chip reactors in which MNPs can be trapped by external permanent magnets at predesigned positions and reagents are flowed through the MNP-filled microchambers. It was feasible to bind enzymes to MNPs and use them in magnechip reactors [14,26,27,28]. Previously, phenylalanine ammonia-lyase (PAL) was immobilized on MNPs coated with an aminopropyl group-modified silica shell. The prepared PAL biocatalyst was used to convert phenylalanine and five other unnatural analogues to their respective arylacrylate derivative in a microfluidic magnechip reactor under continuous flow conditions. The catalytic activity of the PAL biocatalyst did not decrease, even after 14 h of continuous operation [14,26].

**Figure 1:**
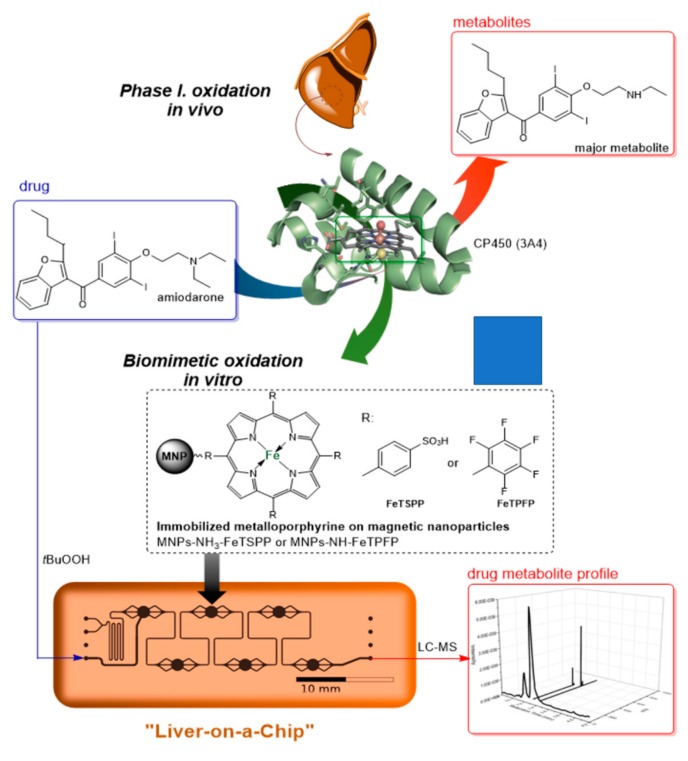
Biomimetic oxidation of amiodarone catalyzed by metalloporphyrines immobilized on magnetic nanoparticles in continuous-flow magnetic chip reactor.

Combining the two above-mentioned methods, a new technique can be introduced. With the usage of synthetic metalloporphyrins bound to MNPs, a system can be established that can mimic the liver on a chip (Figure 1). In this study, two metalloporphyrin derivatives were immobilized onto MNPs via either ionic or covalent interactions. The immobilized catalysts were integrated in a microfluidic magnetic chip reactor and the biomimetic oxidation of an antiarrhythmic drug, amiodarone was investigated.

## 2. Materials and Methods 

### 2.1. Materials

All solvents used in this study were of analytical grade. Methanol (MeOH), trifluoroacetic acid (TFA) and acetic acid were purchased from Merck Ltd. (Budapest, Hungary). Water was obtained from a Millipore (Bedford, MA, USA) Milli-Q water-purification system and used for the preparation of all aqueous solutions. Oxidizing agent *t*-butyl hydroperoxide (*t-*BuOOH was purchased from Sigma-Aldrich (St. Louis, MO, USA). Metalloporphyrines such as 5,10,15,20-tetrakis(2,3,4,5,6-pentafluorophenyl)iron(II) porphyrin (FeTPFP) and 5,10,15,20-tetrakis-(4-sulfonatophenyl)iron(II) porphyrin (FeTSPP) were purchased from Frontier Scientific (Logan, UT, USA). Amiodarone, tris(hydroxymethyl)aminomethane hydrochloride (Tris HCl), MgCl_2_, glucose-6-phosphate, glucose-6-phosphate dehydrogenase, sodium acetate, potassium chloride (KCl) and NADPH were purchased from Sigma-Aldrich. Human liver microsomes pooled from mixed gender was obtained from Sekisui XenoTech Llc. (Kansas City, KS, USA). Magnetic nanoparticles with aminopropyl functions (MNPs-NH_2_) were product of SynBiocat Llc. (Budapest, Hungary).

### 2.2. Methods

#### 2.2.1. HPLC-DAD-MS Analysis

Experiments were carried out on an Agilent 1200 liquid chromatography system coupled with an 6410 QQQ-MS (Agilent Technologies, Palo Alto, CA, USA), equipped with a vacuum degasser, a binary pump, mixer assembly, an auto sampler, a column temperature controller and a diode array detector. Analysis was performed at 45 °C on a Kinetex EVO C_18_ column (50 × 3 mm, 2.6 µm) (Phenomenex), with a mobile phase flow rate of 1.45 mL/min. Composition of eluent A was 0.1% (V/V) trifluoroacetic acid (TFA) in water (pH 1.9), eluent B was a mixture of acetonitrile and water in 95:5 (V/V) with 0.1% (V/V) TFA. A linear gradient of 2–100% B was applied at a range of 0–4.9 min, then 100% B at 4.9–6.0 min. It was followed by a 1.20 min equilibration period prior to the next injection. The injection volume was set at 5 µL and the chromatographic profile was registered at 220 ± 4 nm. The mass spectrometer detector (MSD) operating parameters were as follows: electrospray ionization (ESI) positive ionization, scan ion mode (100–900 m/z), drying gas temperature 350 °C, nitrogen flow rate 11 L/min, nebulizer pressure 40 psi, quadrupole temperature 100 °C, capillary voltage 4000 V, fragmentor voltage 135 V. The results of HPLC-DAD/MS analysis can be found in Supplementary Materials (Supplementary Materials as Figures S1–S21).

#### 2.2.2. Dynamic Light Scattering (DLS) Analysis

Particle size distribution of amino-functionalized MNPs (MNPs-NH_2_) and FeTPFP or FeTSPP porphyrin immobilized on amino-functionalized MNPs (MNPs-NH-FeTPFP or MNPs-NH_3_-FeTSPP) was characterized by dynamic light scattering (DLS, Brookhaven BI-200SM Laser Light Scattering Instrument, Holtsville, NY, USA). The samples were sonicated in methanol for 20 min, then analyzed by a laser beam (λ = 488 nm) at 25 °C in three parallel runs.

#### 2.2.3. ζ-Potential Analysis

The zeta potential of amino-functionalized MNPs (MNPs-NH_2_) and FeTPFP or FeTSPP porphyrin immobilized on amino-functionalized MNPs (MNPs-NH-FeTPFP or MNPs-NH_3_-FeTSPP) was measured with Zeta Potential Analyzer (Brookhaven) using the Zeta PALS (Phase Analysis Light Scattering) method. Re-dispersed samples were diluted 5-fold in 1 mM KCl aqueous solution. Measurements carried out in a disposable, solvent resistant micro cuvette took 2 min. Zeta potential was calculated from the electrophoretic mobility using Smoluchowski equation.

#### 2.2.4. Covalent Immobilization of FeTPFP on Functionalized Magnetic Nanoparticles (MNPs)

Amino-functionalized magnetic nanoparticles (MNPs-NH_2_, 10 mg) was sonicated in diglyme (400 µL) for 10 min. Then a solution of 5,10,15,20-tetrakis(2,3,4,5,6-pentafluorophenyl)iron(II) porphyrin (FeTPFP) in diglyme (600 µL, 2.5 mg/mL) was added to the suspension and the mixture was shaken for 72 h at 60 °C. After magnetic separation, the MNPs were washed with isopropanol, distilled water, and methanol and were dried in vacuum cabinet for 4 h.

#### 2.2.5. Ionic Immobilization of FeTSPP on Functionalized MNPs

Amino-functionalized magnetic nanoparticles (MNPs-NH_2_, 10 mg) were added to methanol:sodium acetate buffer (4:1 V/V, 8 mL, pH = 4.5) and sonicated for 10 min. Then a solution of 5,10,15,20-tetrakis-(4-sulfonatophenyl)iron(II) porphyrin in acetate buffer (4:1 V/V, 8 mL, pH = 4.5 (750 µL, 1.0 mg/mL) was added to the mixture and the suspension was shaken for 5 min at room temperature. After magnetic separation, it was washed with methanol, then dried in vacuum cabinet for 4 h.

#### 2.2.6. Immobilization Yield (*Y_I_*) of MNP-Porphyrines

After the immobilization of FeTPFP or FeTSPP metalloporphyrin, a sample (900 μL) taken directly from the residual binding solvent freed from MNPs was analyzed by a Genesys 2 type ultraviolet-visible (UV-VIS) spectrophotometer (Thermo Fisher Scientific Inc., Waltham, MA, USA) at room temperature. The specific wavelength (λ_max_) of the corresponding metalloporphyrin was determined (λ_max_ = 407 nm for FeTPFP and λ_max_ = 395 nm for FeTSPP), then calibration curves were also recorded. Immobilization yield (*Y_I_*, %) was calculated from the following:

$$Y_{I}=\frac{c_{2P}}{c_{1P}}\times 100$$

where *c*_1*P*_ is the initial porphyrin concentration, *c*_2*P*_ is the residual porphyrin concentration in the binding solution.

#### 2.2.7. Metabolism of Amiodarone (**1**) by Human Liver Microsomal Reactions

Sodium pyrophosphate (125 µL, 6.38 mg/mL), magnesium chloride (50 µL, 3 mM), glucose-6-phosphate (25 µL, 13 mg/mL), glucose-6-phosphate dehydrogenase (25 µL, 20 IU/mL), Tris-HCl buffer (170 µL, 15.76 mg/mL), human liver microsome (50 µL, final concentration is 1000 µg/mL) and amiodarone solution (in methanol, 5 µL, 0.65 mg/mL) was pipetted in an Eppendorf tube. It was held at 37 °C for 5 min. After that NADPH (50 µL, 3.72 mg/mL) was added to the reaction mixture. It was shaken for 30 min at 37 °C. The reaction was stopped with the addition of methanol (0.5 mL, −20 °C). The microsomes were separated by ultracentrifugation (at 10,000 g, for 5 min). The upper clear phase (0.8 mL) was analyzed by HPLC-DAD-MS method described in Section 2.2.1.

#### 2.2.8. General Method of Homogeneous Biomimetic Batch Reactions of Amiodarone (**1**)

Amiodarone solution (50 µL, 5.7 mg/mL in methanol:sodium acetate buffer, 4:1 V/V, pH = 4.5, 64 mM), solvent completion (150 µL, methanol:sodium acetate buffer, 4:1 V/V, pH = 4.5), porphyrin solution (50 µL, 0.9 mg/mL in methanol:sodium acetate buffer, 4:1 V/V, pH = 4.5, 64 mM) and oxidizing agent solution (*t-*BuOOH, 50 µL, 88.2 mM in methanol:sodium acetate buffer, 4:1 V/V, pH = 4.5, 64 mM) was pipetted in an Eppendorf tube. It was shacked for 1 h at room temperature at 400 rpm. The reaction mixture (300 µL) was analyzed by HPLC-DAD-MS method described in Section 2.2.1.

#### 2.2.9. General Method of Heterogeneous Biomimetic Batch Reactions of Amiodarone (**1**)

In Eppendorf tubes porphyrin loaded MNPs (MNPs-NH-FeTPFP or MNPs-NH_3_-FeTSPP; 2 mg) and amiodarone solution (0.5 mL, 2 mg/mL in methanol:sodium acetate buffer 4:1 V/V, pH = 4.5, 64 mM) was sonicated for 20 min. The concentration was adjusted to 1 mg/mL of amiodarone. The reaction was started with the addition of the oxidizing agent (*t-*BuOOH, 5 molar equivalent). The reaction mixtures were shaken for 1 h at room temperature. After magnetic separation the clear phase (1 mL) was analyzed by HPLC-DAD-MS technique described in Section 2.2.1.

#### 2.2.10. Calculation of the Biocatalytic Parameters

Conversion of the substrate (*c*, %), biocatalytic activity (*U*_B_, U/g), specific activity (*U*_P_, U·g^−1^), turnover frequency (TOF, mol_prod_·mol_cat_^−1^·h^−1^) and space time yield (STY, mg·L^−1^·h^−1^) were calculated by using the following equations based on HPLC chromatograms:

(1) $$c\;\left(\%\right)=100-\frac{n_{S}}{n_{S}+n_{P}}\times 100$$

where *n*_S_ and *n*_P_ are the molar amounts of substrate (*S*) and product(s) (*P*),

(2) $$U_{B}\left(U/g\right)=\frac{\left(n_{S}\times c\right)}{\left(t\times m_{B}\right)}$$

where *U* is rate of the substrate conversion (µmol/min), *t* is reaction time, *m*_B_ is the mass of the biocatalyst,

(3) $$U_{P}\left(U/g\right)=\frac{\left(n_{S}\times c\right)}{\left(t\times m_{P}\right)}$$

where *m*_P_ is the mass of the porphyrin in the immobilized biocatalyst,

(4) $$TOF\left(mol/\mathrm{mol}\times h\right)=\frac{n_{products}}{n_{catalyst}}\times \frac{1}{t}$$

(5) $$STY\left(\frac{mg}{l}\times h\right)=\frac{m_{p}}{V_{r}\times t}$$

where *m_p_* is the mass of the products in mg, *V_r_* is the volume of the reactor and *t* is time.

#### 2.2.11. General method for Loading the Microfluidic Magnetic Chip Reactor with Catalyst

Porphyrin-loaded MNPs (6 mg) were added to methanol:sodium acetate buffer (4:1 V/V, pH = 4.5, 64 mM, 2 mL). After sonication, the suspension was loaded in a syringe and was driven through a microfluidic chip to fill up the chambers with nanoparticles following the previously described method, [14,26] outlined here shortly. During the filling of the chip, the MNPs were accumulated in the reaction chambers (diameter 3600 µm, height 110 µm) due to the magnetic field of the permanent magnets located beneath the chambers. Once the last chamber was saturated the process was repeated with the forthcoming upstream chamber until all chambers were filled up.

#### 2.2.12. General Method of Microfluidic Biomimetic Reactions

**Figure 2:**
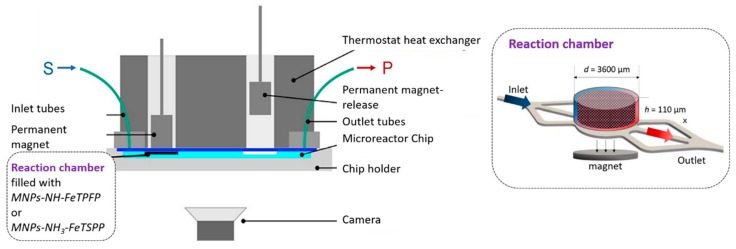
Cross-sectional view of Magneflow chip holder with the microreactor chip. A layer of magnetic nanoparticles (MNP) is formed in the reaction chambers due to the magnetic field applied by moving the permanent magnets toward the microreactor chip. Layer consistency is assessed through the camera image; reactor temperature is maintained by the thermostat heat exchanger.

Biomimetic reactions were carried out by using Magneflow system (SpinSplit LLC, Budapest, Hungary) as follows. Amiodarone (0.5 mg/mL in methanol:sodium acetate buffer, 4:1 V/V, pH = 4.5) and oxidizing agent (5 equiv., 7.35 mM in methanol:sodium acetate buffer, 4:1 V/V, pH = 4.5, 64 mM) solution were driven through the MNP loaded chip reactor ‘MagneChip 6/3′ (SpinSplit LLC) at four different flow rates (15, 30, 45 and 60 µL /min) following a pre-programmed sequence in SpinStudio (SpinSplit LLC) software. A timeframe of 15 min was provided to ensure the reach of equilibrium before samples were taken. The samples were analyzed by HPLC-DAD-MS technique (Figure 2).

## 3. Results and Discussion

### 3.1. Immobilization of Metaloporphyrin on Functionalized Magnetic Nanoparticles

Two species of synthetic metalloporphyrin (FeTPFP and FeTSPP) were immobilized on aminopropyl-grafted magnetic nanoparticles (MNP-TEOS-NH_2_) by two different ways. FeTPFP was immobilized by covalent binding based on aromatic nucleophilic substitution between the pentafluorophenyl ring of the porphyrin and amino-group of MNPs. FeTSPP was attached to the MNP surface by ionic interactions between the sulfuric groups of porphyrin and amino-groups of MNPs. To characterize the efficiency of the functionalized MNPs in immobilization of the two type of metalloporphyrins the metalloporphyrin content of the residual binding solution was determined by UV-VIS spectroscopy. Based on the determination of immobilization yields (*Y_I_*), the FeTPFP content of the FeTPFP-MNPs was 0.054 mg/mg catalyst. In case of FeTSPP, the metalloporphyrin content was 0.075 mg/mg immobilized catalyst. These differences can be explained by the rapid and mild binding by ionic forces, in contrast to covalent binding which require more energy.

Colloidal stability of a dispersion consisting nanoparticles is usually a key issue, because the constant stability of the individual particles against aggregation and sedimentation is essential in efficient transport processes, which is essential in heterogenic catalytic reactions. Stability of colloidal systems can be predicted by measuring the zeta potential. The higher absolute value of zeta potential suggests increased stability of the colloidal dispersion and lower tendency towards coagulation or flocculation (−5 mV < ζ < 5 mV brings about rapid coagulation or flocculation) [29]. The zeta potential of all three derivatized MNPs were between −5 and −23 mV, indicating considerable colloidal stability. The largest absolute value (more charged surface) was observed for the amine functionalized particles, while shielding the surface charge either with covalent binding or ionic interactions resulted in lower absolute values of zeta potential. In accordance with the immobilization yields, modification with FeTSPP caused a drop in the zeta potential to an absolute value of around 5 mV. Comparison of the amino-functionalized MNPs (MNPs-NH_2_) and the two kinds of porphyrin-covered MNPs (MNP-NH-FeTPFP and MNP-NH_3_-FeTSPP) revealed that the hydrodynamic diameters of porphyrin-modified MNPs were smaller than that of the amino-functionalized MNPs. This means, that even after ultra-sonication the amino-functionalized MNPs tend to aggregate more, because the functional groups on their surface were more ionizable. After immobilization of the metalloporphyrins, most of the ionizable amino functions on the surface were reacted, therefore less ionization could take place (Table 1).

### 3.2. Metabolism of Amiodarone (**1**)

**Figure 3:**
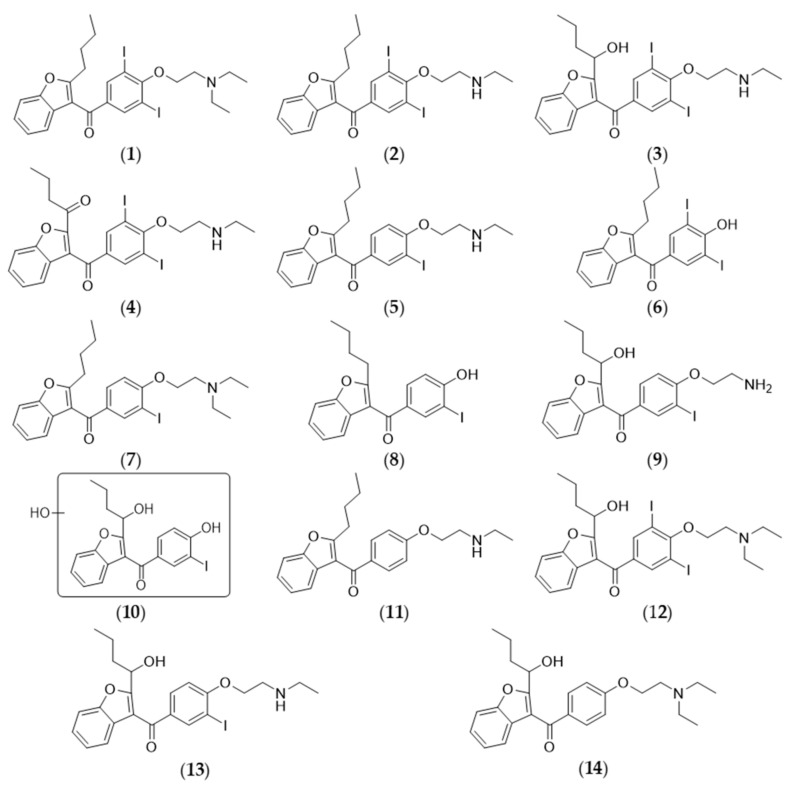
Amiodarone (**1**) and the detected derivatives (the assumed structures were based on their HRMS signal) of amiodarone (**2**–**14**).

Amiodarone antiarrhythmic drug was used as model substrate for investigation the biomimetic oxidation catalyzed by metalloporphyrin. In the human body, the CP450-catalyzed oxidation of amiodarone produces *N*-deethyl-amiodarone (**2**) as the major human metabolite, however secondary deethylation resulting primary amine, mono and di-deiodinated, *O*-dealkylated and hydroxylated derivatives (**12**) have been also demonstrated at very low quantity [30,31,32]. In our recently published study, new derivatives were formed and identified in biomimetic oxidation [22]. The possible structures of metabolites (**2**–**12**) of amiodarone (**1**) were deduced from HRMS measurements (Figure 3).

The structures of the derivatives (**2**–**14**) from amiodarone (**1**) indicate, that the oxidative metabolism of amiodarone happened by *N*- and *O*-dealkylations, dehalogenations, hydroxylations or oxidations.

### 3.3. Biomimetic Oxidation of Amiodarone (**1**) Catalyzed by Metalloporphyrins in Batch Mode

To study the effect of immobilization on the catalytic properties of metalloporphyrines, heterogeneous and homogeneous biomimetic reactions in batch mode were compared under the same conditions (Table 2). Two different synthetic metalloporphyrines (FeTPFP and FeTSPP) were used in our experiments in non-immobilized soluble form (as homogenous reactions) and in immobilized form on MNPs (as heterogeneous reactions). In case of homogenous oxidations, the metabolite profiles of the two metalloporphyrin catalysts were different. With FeTPFP as catalyst, *O*-dealkylation and *N*-deethylation were the main reactions along with the formation of monodehalogenated-amiodarone (**7**) as minor product. With FeTSPP as catalyst, *N*-deethylation, dehalogenation and hydroxylation were the most pronounced and oxo-amiodarone (**4**) was formed from hydroxy-amiodarone (**3**). After immobilization on MNPs the metabolite profile of the metalloporphyrines changed significantly. The same *N*-deethyl-amiodarone (**2**) was formed as main metabolite as in the homogeneous batch reaction, while no further derivatives that formed in the homogeneous reaction (**3**, **4**, and **5**) were generated in the heterogeneous reaction. This means that by immobilization the hydroxylation of monodeethyl-amiodarone become unfavored reaction. In the heterogeneous reaction using immobilized FeTSPP catalyst, novel metabolites were formed involving *O*-dealkylated-amiodaron and further products by subsequent dehalogenations, dealkylations and hydroxylations.

The biocatalytic activity (*U*_B_) and specific biocatalytic activity (*U*_P_) are key parameters of a catalyst: the *U*_B_ shows the productivity referring to the mass unit (usually in g) of the total catalyst (non- and immobilized as well) and *U*_P_ vale refers to the “active” porphyrin content (usually in g) of the immobilized catalyst. Results in Table 3 shows, that every biomimetic oxidation catalyzed by a metalloporphyrin catalyst provided much higher catalytic activity, than the human liver microsomes-based system. The *U*_B_ (and *U*_P_ as well) values of the two different metalloporphyrines (FeTPFP and FeTSPP) were comparable in homogenous reaction media, but after immobilization on MNPs significant differences between the *U*_B_ (and *U*_P_) values with the two porphyrin could be observed. This result can be explained by the different immobilization yields, binding forces and topological characters of the immobilized catalysts. The increase in specific catalytic activity (*U*_P_) of MNPs-NH-FeTPFP catalyst can be caused by the good dispersion and stabilization of the porphyrins on to MNPs surface.

### 3.4. Biomimetic Oxidation of Amiodarone (**1**) Catalyzed by MNP-Porphyrines in Continuous-Flow Chip Reactor—Liver-on-a-Chip

Generic PDMS microfluidic chip was used in our experiments, with 6 reaction chamber. After loading the chip, each reaction chamber contained about 250 µg catalyst according to resonance frequency shift measurement based on our previous study [28]. The metabolite profile from chip reactor was different from the batch reactions, because two novel derivatives (**13**, **14**) appeared at significant quantity. The metabolite profile of the two investigated metalloporphyrines (FeTSPP and FeTPFP) were quite similar. The effect of the flow rate could be clearly recognized: The faster the flow rate of amiodarone solution, the less metabolite was formed (Table 4).

**Figure 4:**
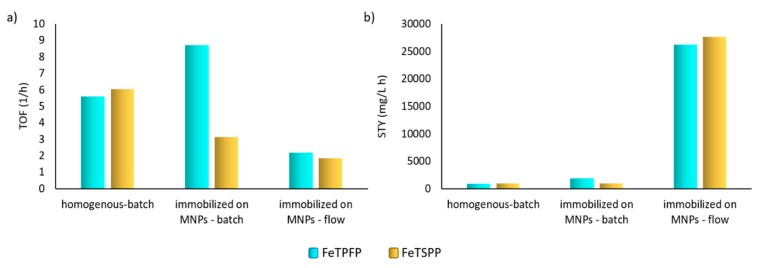
Turnover frequency (TOF) (**a**) and space time yield (STY) (**b**) values of metalloporphyrin (FeTPFP or FeTSPP) catalysts in their soluble (homogenous) or MNP-immobilized (heterogeneous) forms in biomimetic oxidation of amiodarone (**1**) in batch mode or continuous-flow magnetic chip reactor.

To characterize the efficiency of the flow-chip reactor, the turnover frequency (TOF) and the space time yield (STY) values were calculated and compared to batch systems (Figure 4). The turnover values—characterizing the catalyst activity by the number of moles substrate transformed by one mole of catalyst per hour—were in the same range, between 2 and 9, with each of the porphyrin catalyst consistently to the *U*_P_ values in Table 3. Notably, the STY—indicating the volumetric productivity of a reactive system—of the microfluidic magnetic chip reactor was remarkably higher than any of the biomimetic oxidation in batch mode.

## 4. Conclusions

The investigation of drug metabolites is a key issue during the early stage drug discovery; especially liver related first phase oxidative metabolism catalyzed by CP450 enzymes has a great importance. Traditional animal experiments in vivo or in vitro methods or experiments with liver cells or its microsomes; although they are accepted by FDA, have many limitations, which complicate the effective metabolite production already at analytical scale too. Synthetic metalloporphyrines have high similarity to the active site of CP450, thus they are able to mimic the enzymatic reactions without the complex biological matrix. This study showed that immobilization of metalloporphyrines onto surface-functionalized magnetic nanoparticles can provide biocatalyst with increased activity in the biomimetic oxidation of amiodarone. The type of the metalloporphyrin, the way of immobilization and mode of the process are able to influence the metabolite profile. Integration of these immobilized metalloporphyrines into microfluidic magnetic chip reactors demonstrated that drug metabolites can be produced efficiently in extremely small reactor volumes resulting in excellent volumetric productivity. In addition, not only the in vivo major metabolite can be easily synthetized by the microfluidic magnetic “Liver-on-a-chip” system, but new derivatives can be produced opening up unique novel opportunities for modern drug discovery.

## Figures and Tables

**Table 1:** Dynamic light scattering (DLS) and ζ-potential data of the modified MNPs.

Type of MNPs	*d*_P_ (nm)	ζ-Potential (mV)
MNPs-NH_2_	429 ± 46	−22.9 ± 0.8
MNPs-NH-FeTPFP	336 ± 25	−16.0 ± 0.8
MNPs-NH_3_-FeTSPP	296 ± 4	−5.3 ± 1.2

**Table 2:** Metabolite profile of amiodarone from homogenous (free metalloporphyrin catalyzed: FeTPFP or FeTSPP) and heterogeneous (immobilized metalloporphyrin on MNPs: MNPs-NH-FeTPFP or MNPs-NH_3_-TSPP) biomimetic oxidations in batch mode.

Metabolite ^a^	In Vitro Human Liver Microsomal Investigation	Homogenous Biomimetic Reaction *“Free Metalloporphyrin”*		Heterogeneous Biomimetic Reaction *“Immobilized Metallo-porphyrin on MNPs”*	
		FeTPFP	FeTSPP	MNPs-NH-FeTPFP	MNPs-NH_3_-FeTSPP
amiodarone (**1**)	86.4	8.1	4.3	6.3	53.0
(**2**)	13.3	66.7	66.5	62.6	38.0
(**3**)	-	-	1.0	-	-
(**4**)	-	-	7.6	-	-
(**5**)	-	-	10.5	1.4	1.4
(**6**)	-	24.3	-	25.7	5.9
(**7**)	-	0.9	-	-	-
(**8**)	-	-	-	-	1.3
(**9**)	-	-	-	-	0.4
(**10**)	-	-	-	-	0.1
(**11**)	-	-	-	-	-
(**12**)	0.3	-	-	-	-
(**13)**	-	-	-	-	-
(**14**)	-	-	-	-	-
other ^b^	-	-	10.0	4.0	-

^a^ the structure and the amount of metabolites were determined by LC-MS measurement, where the ratio of metabolites based relative peak area (%) at λ = 220 ± 4 nm on DAD-chromatograms, ^b^ not identified compounds under limit of detection.

**Table 3:** Biocatalytic activity (*U*_B_) and specific biocatalytic activity (*U*_P_) of human liver microsomes (HLM) and metalloporphyrins (FeTPFP or FeTSPP) in their soluble (homogenous catalytic reaction) and MNP immobilized (heterogeneous catalytic reaction) forms in biomimetic oxidation of amiodarone (**1**).

Activity	Homogenous Catalytic Reaction			Heterogenous Catalytic Reaction	
	HLM	FeTPFP	FeTSPP	MNPs-NH-FeTPFP	MNPs-NH_3_-FeTSPP
*U*_B_ (U/g)	0.5	132.7	153.6	12.1	6.1
*U*_P_ (U/g)	-	132.7	153.6	223.8	80.8

**Table 4:** Metabolite profile of amiodarone from immobilized metalloporphyrin (MNPs-NH-FeTPFP or MNPs-NH_3_-TSPP) catalyzed biomimetic oxidation in continuous-flow magnechip reactor–“Liver-on-a-Chip”.

Metabolites ^a^	Flow Rate (μL/min)							
	15		30		45		60	
	FeTPFP	FeTSPP	FeTPFP	FeTSPP	FeTPFP	FeTSPP	FeTPFP	FeTSPP
amiodarone (**1**)	61.0	58.8	75.7	77.0	84.5	84.4	88.4	87.9
(**2**)	0.5	-	0.5	0.3	0.6	0.4	0.8	0.6
(**11**)	10.8	11.5	5.6	5.6	2.7	2.7	1.1	2.2
(**13**)	21.5	23.2	13.4	12.9	8.2	8.5	6.3	6.9
(**14**)	3.9	4.4	3.3	3.0	2.6	2.9	2.3	1.8
other ^b^	2.3	2.2	1.5	1.4	1.3	1.2	1.2	0.7

^a^ the structure and the amount of metabolites were determined by LC-MS measurement, where the ratio of metabolites based relative peak area (%) at λ = 220 ± 4 nm on DAD-chromatograms, ^b^ not identified compounds under limit of detection.

## Supplementary Materials

The following are available online at https://www.mdpi.com/2072-666X/10/10/668/s1, Figure S1: Representative HPLC/MS data of human liver microsomal investigation of amiodarone, Figure S2: Representative HPLC/MS data of non-immobilized FeTPFP-catalyzed biomimetic oxidation of amiodarone, Figure S3: Representative HPLC/MS data of non-immobilized FeTSPP-catalyzed biomimetic oxidation of amiodarone, Figure S4: Representative HPLC/MS data of FeTPFP-functionalized magnetic nanoparticles-catalyzed biomimetic oxidation of amiodarone in batch mode, Figure S5: Representative HPLC/MS data of FeTSPP-functionalized magnetic nanoparticles-catalyzed biomimetic oxidation of amiodarone in batch mode, Figure S6: Representative HPLC/MS data of FeTPFP-functionalized magnetic nanoparticles-catalyzed biomimetic oxidation of amiodarone in continuous-flow magnetic chip reactor, Figure S7: Representative HPLC/MS data of FeTSPP-functionalized magnetic nanoparticles-catalyzed biomimetic oxidation of amiodarone in continuous-flow magnetic chip reactor, Figure S8: MS spectrum of amiodarone (**1**), Figure S9: MS spectrum of amiodarone metabolite (**2**), Figure S10: MS spectrum of amiodarone metabolite (**3**), Figure S11: MS spectrum of amiodarone metabolite (**4**), Figure S12: MS spectrum of amiodarone metabolite (**5**), Figure S13: MS spectrum of amiodarone metabolite (**6**), Figure S14: MS spectrum of amiodarone metabolite (**7**), Figure S15: MS spectrum of amiodarone metabolite (**8**), Figure S16: MS spectrum of amiodarone metabolite (**9**), Figure S17: MS spectrum of amiodarone metabolite (**10**), Figure S18: MS spectrum of amiodarone metabolite (**11**), Figure S19: MS spectrum of amiodarone metabolite (**12**), Figure S20: MS spectrum of amiodarone metabolite (**13**), Figure S21: MS spectrum of amiodarone metabolite (**14**).
