# Supplementary material for: Liver-on-a-Chip‒Magnetic Nanoparticle Bound Synthetic Metalloporphyrin-Catalyzed Biomimetic Oxidation of a Drug in a Magnechip Reactor
Source: Micromachines (Basel). 2019 Oct 1;10(10):668. doi: 10.3390/mi10100668 (PMC6843572; doi:10.3390/mi10100668)
Supplement: Supplementary file 1 [file micromachines-10-00668-s001.pdf]

micromachines

Supplementary Materials

Liver-on-a-Chip–Magnetic nanoparticle bound synthetic metalloporphyrin-catalyzed biomimetic oxidation of a drug in a magnechip reactor

Balázs Decsi, Réka Krammer, Kristóf Hegedűs, Ferenc Ender, Benjámin Gyarmati, András Szilágyi, Róbert Tőtős, Gabriel Katona, Csaba Paizs, György T. Balogh and László Poppe and Diána Balogh-Weiser

1. Results of HPLC-DAD-MS analysis

1.1. Liquid chromatography (HPLC) chromatograms of the biomimetic reactions

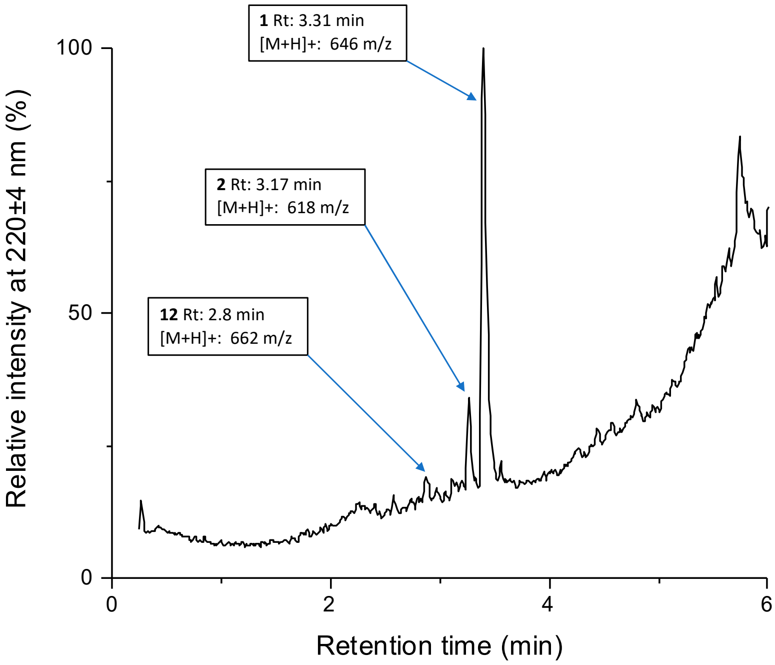

Figure S1. Representative HPLC/MS data of human liver microsomal investigation of amiodarone.

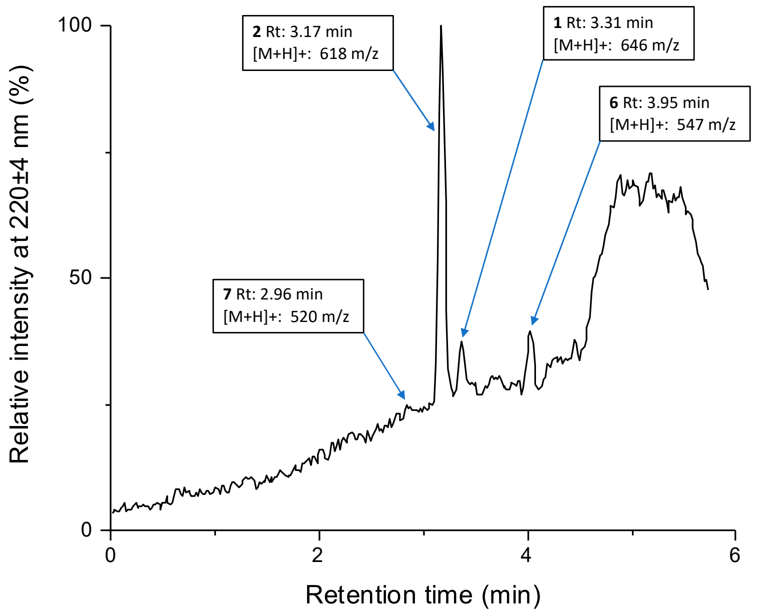

Figure S2. Representative HPLC/MS data of non-immobilized FeTPFP-catalyzed biomimetic oxidation of amiodarone.

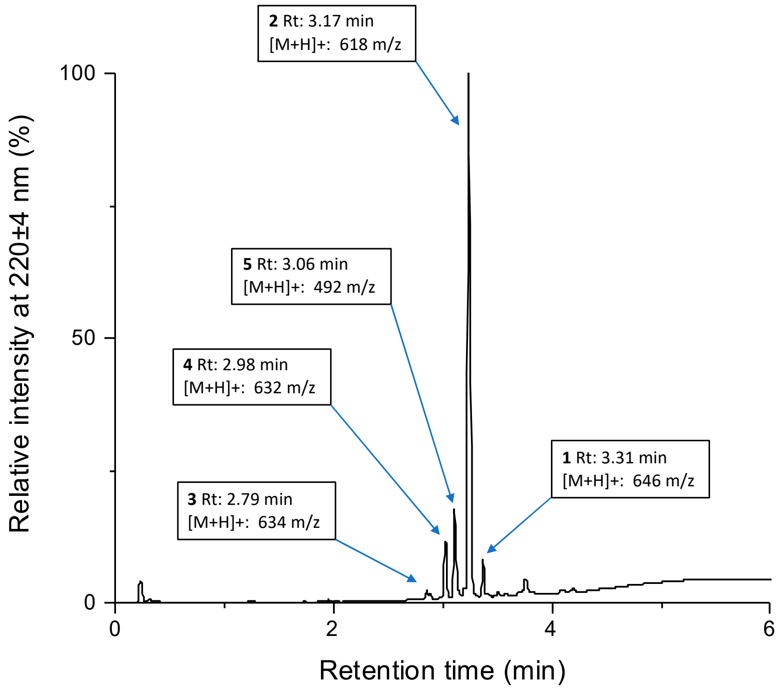

Figure S3. Representative HPLC/MS data of non-immobilized FeTSPP-catalyzed biomimetic oxidation of amiodarone.

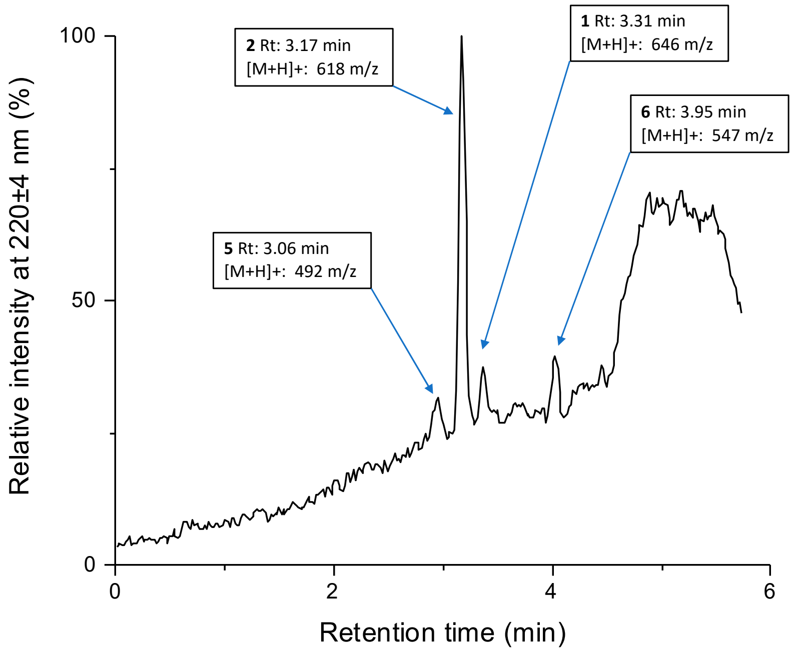

Figure S4. Representative HPLC/MS data of FeTPFP-functionalized magnetic nanoparticles-catalyzed biomimetic oxidation of amiodarone in batch mode.

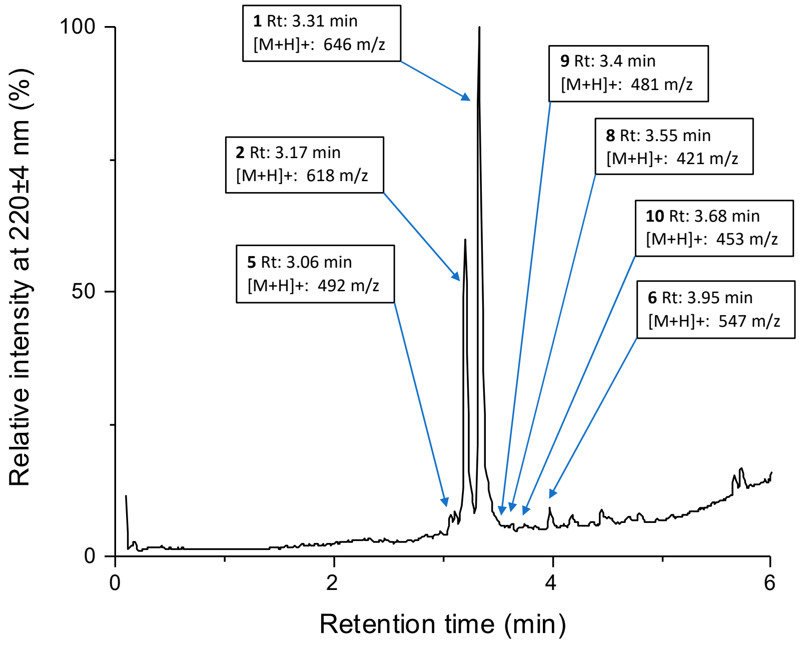

Figure S5. Representative HPLC/MS data of FeTSPP-functionalized magnetic nanoparticles-catalyzed biomimetic oxidation of amiodarone in batch mode.

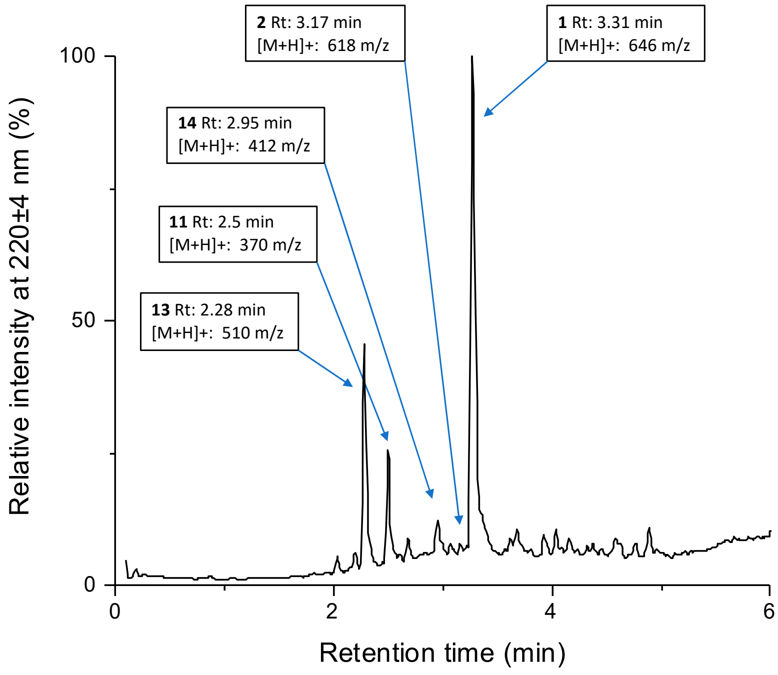

Figure S6. Representative HPLC/MS data of FeTPFP-functionalized magnetic nanoparticles-catalyzed biomimetic oxidation of amiodarone in continuous-flow magnetic chip reactor.

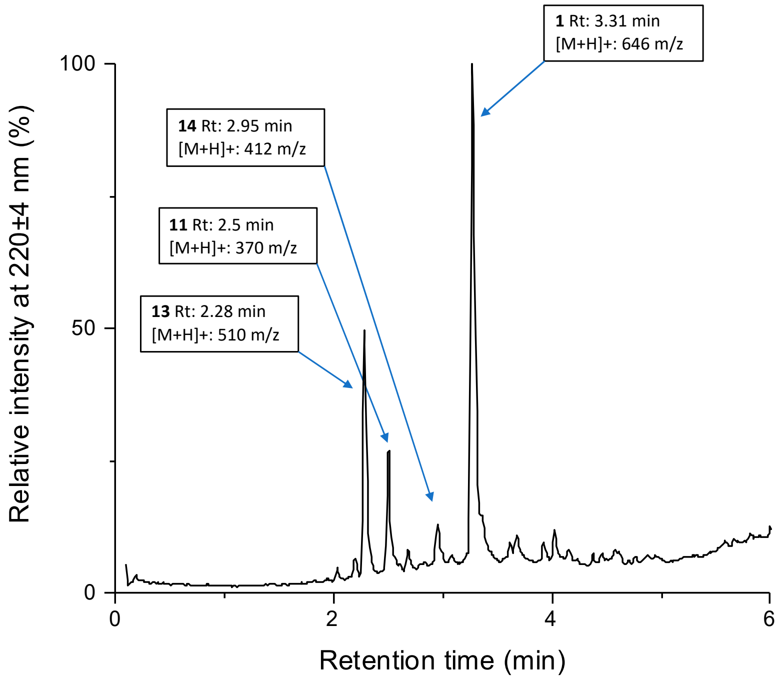

Figure S7. Representative HPLC/MS data of FeTSPP-functionalized magnetic nanoparticles-catalyzed biomimetic oxidation of amiodarone in continuous-flow magnetic chip reactor.

1.2. Identification of amiodarone and its metabolites by mass spectroscopy (MS)

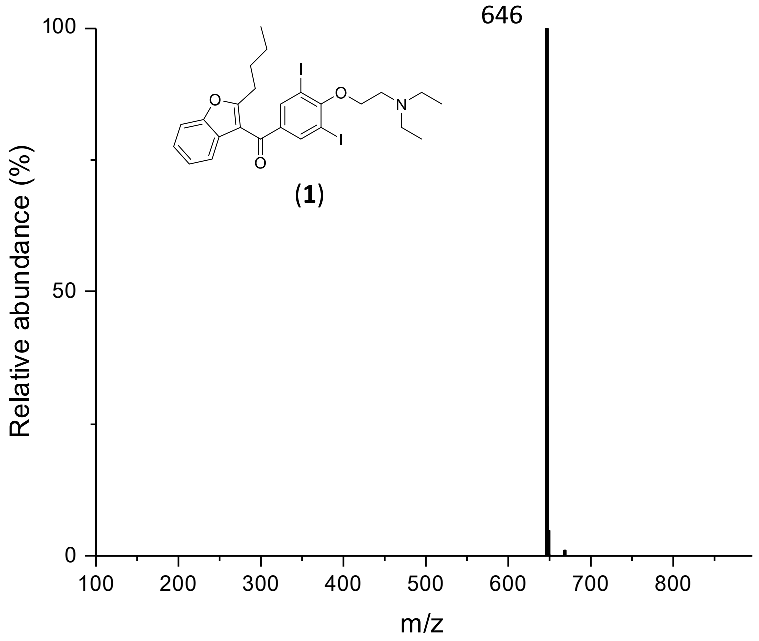

Figure S8. MS spectrum of amiodarone (**1**).

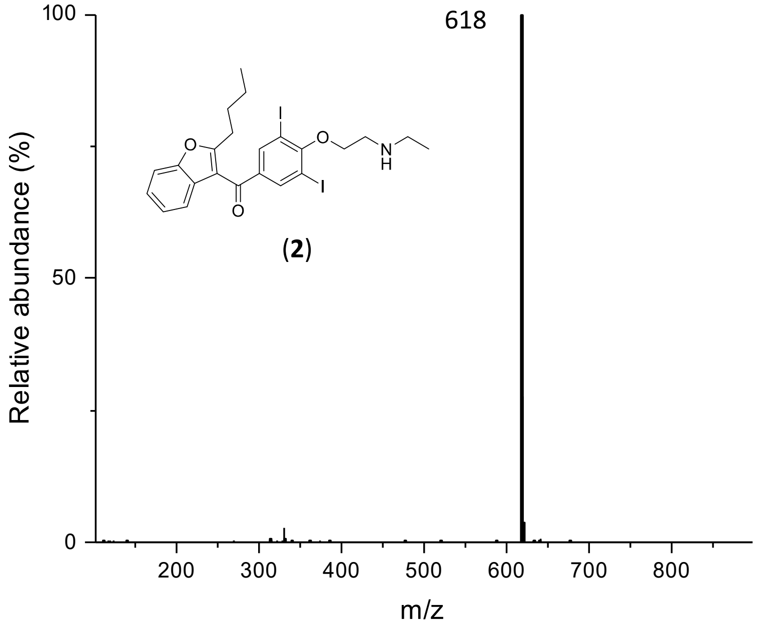

Figure S9. MS spectrum of amiodarone metabolite (**2**).

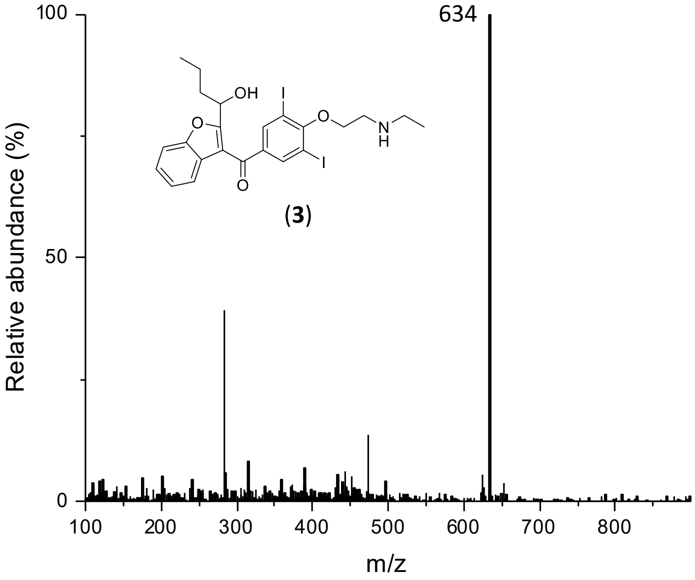

Figure S10. MS spectrum of amiodarone metabolite (**3**).

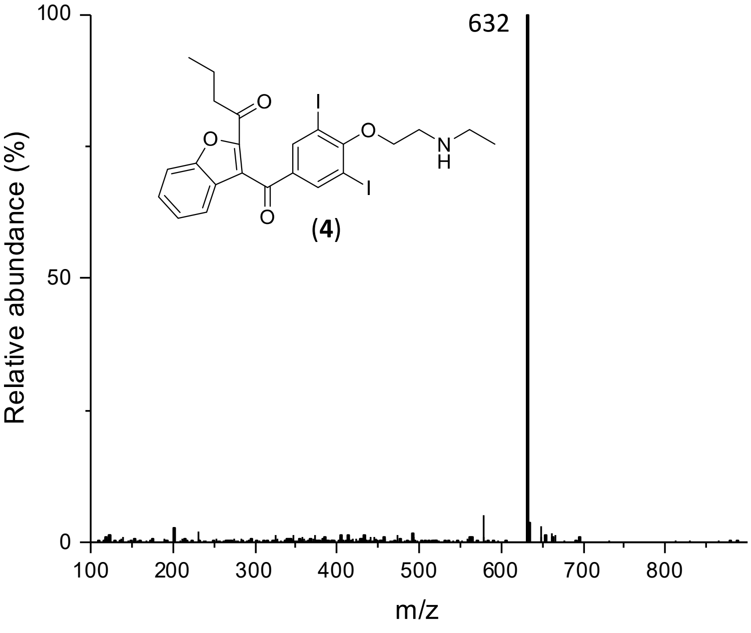

Figure S11. MS spectrum of amiodarone metabolite (**4**).

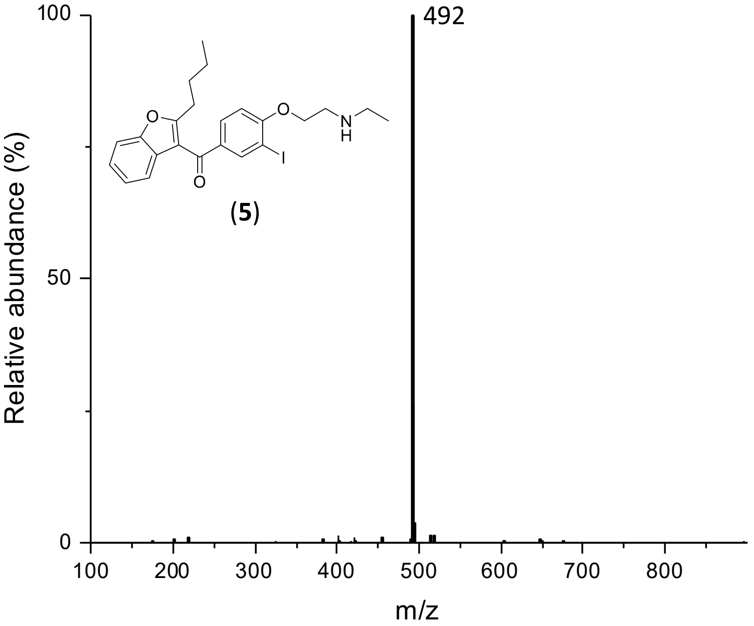

Figure S12. MS spectrum of amiodarone metabolite (**5**).

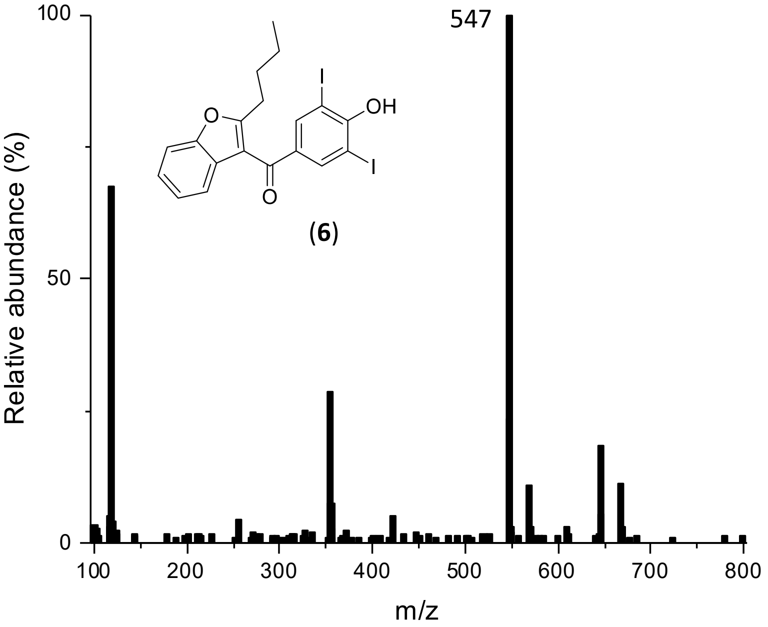

Figure S13. MS spectrum of amiodarone metabolite (**6**).

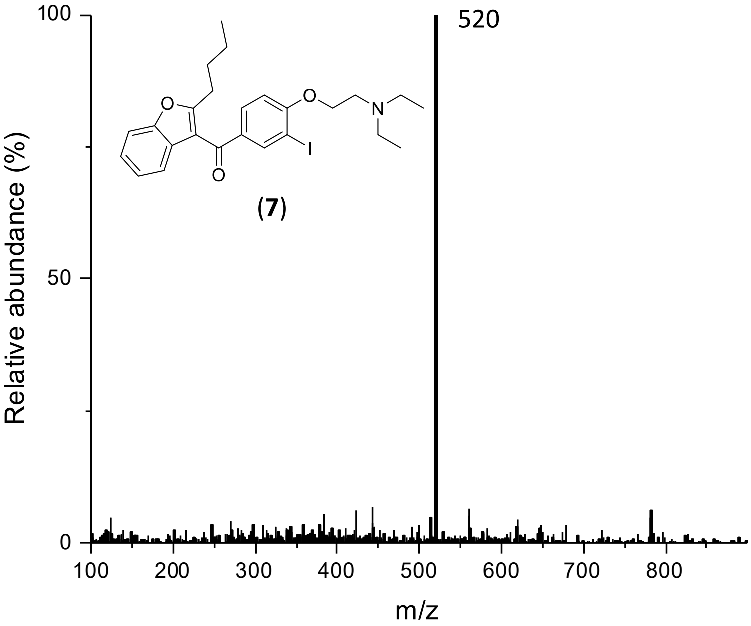

Figure S14. MS spectrum of amiodarone metabolite (**7**).

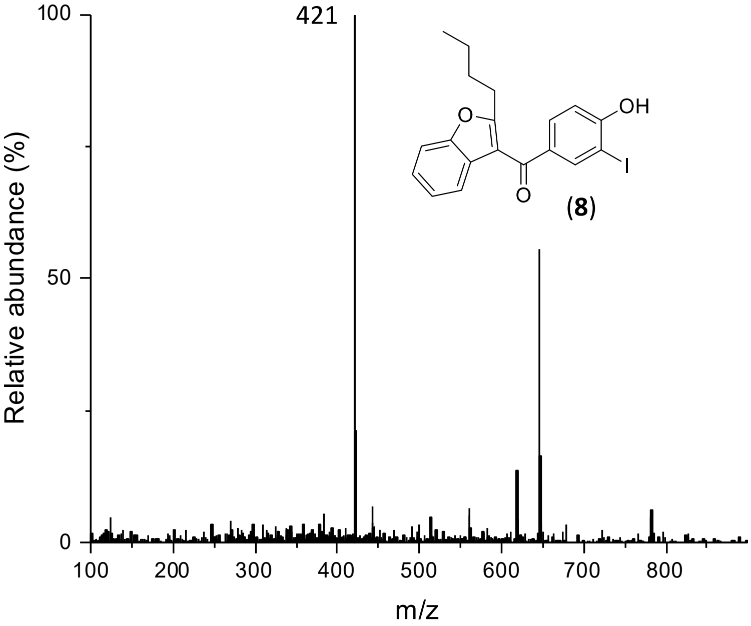

Figure S15. MS spectrum of amiodarone metabolite (**8**).

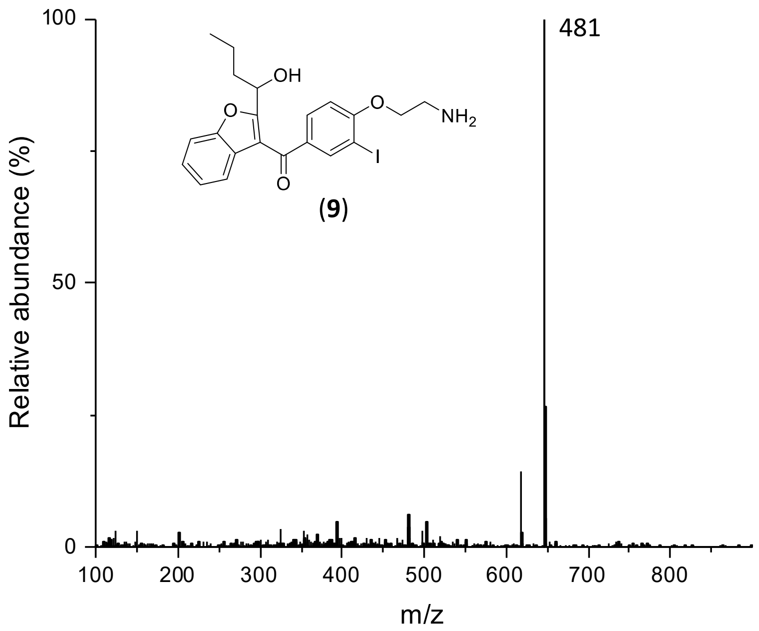

Figure S16. MS spectrum of amiodarone metabolite (**9**).

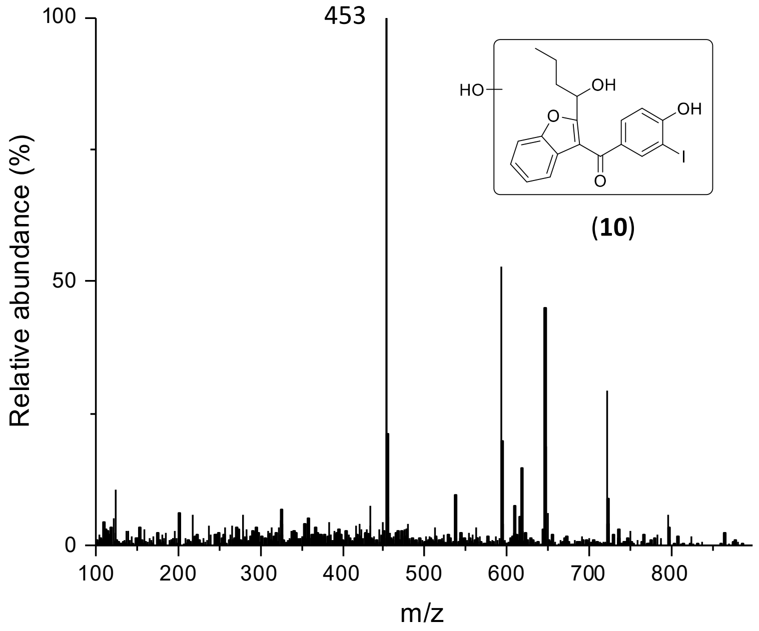

Figure S17. MS spectrum of amiodarone metabolite (**10**).

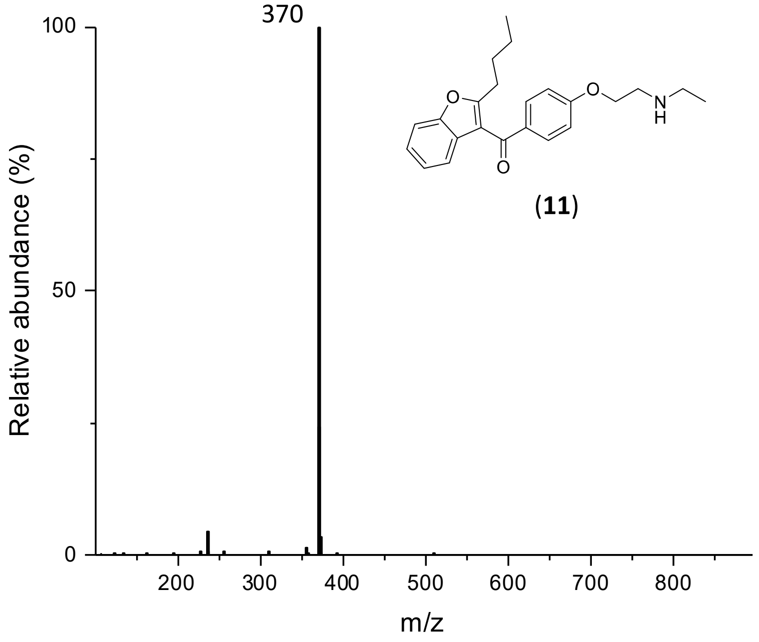

Figure S18. MS spectrum of amiodarone metabolite (**11**).

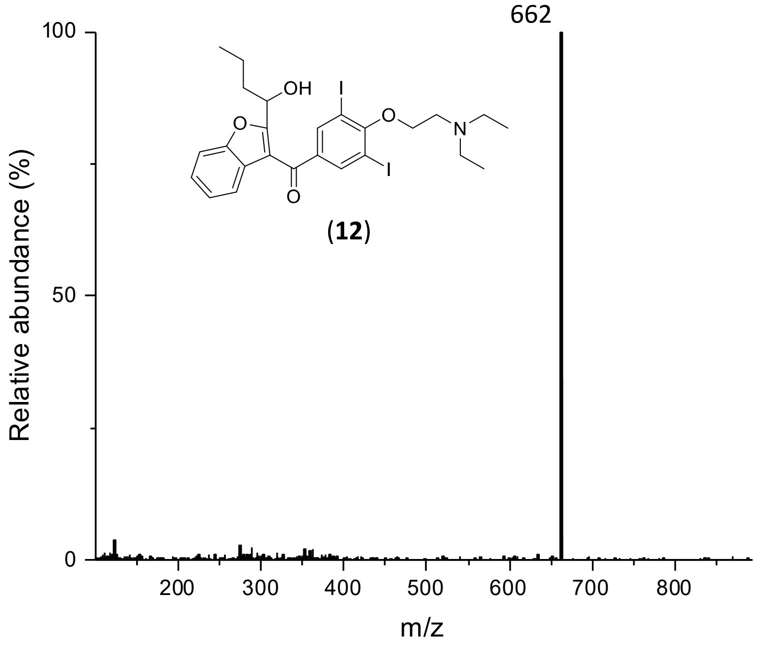

Figure S19. MS spectrum of amiodarone metabolite (**12**).

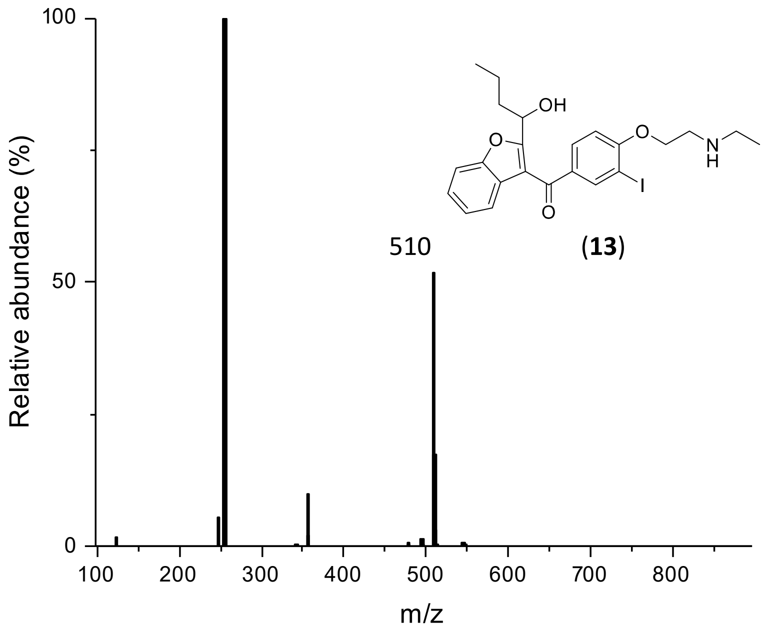

Figure S20. MS spectrum of amiodarone metabolite (**13**).

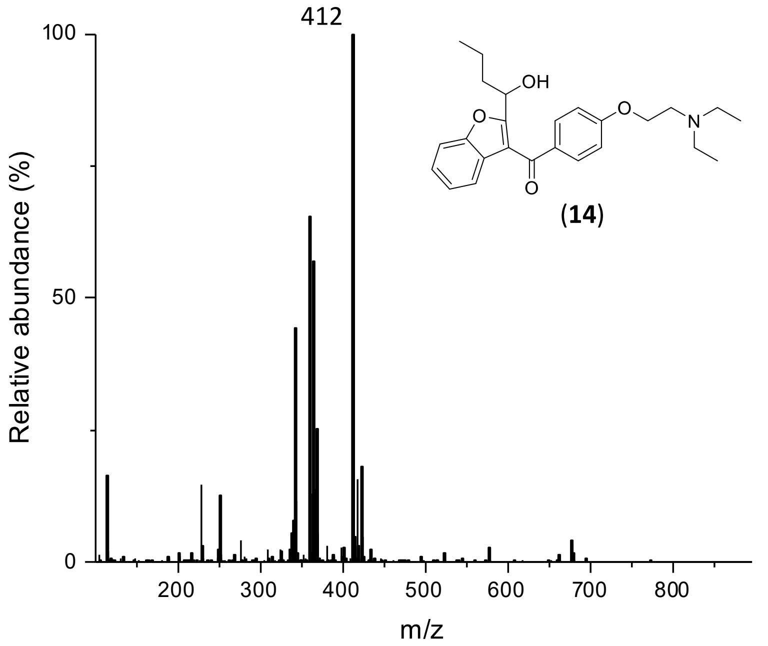

Figure S21. MS spectrum of amiodarone metabolite (**14**).

© 2019 by the authors. Submitted for possible open access publication under the terms and conditions of the Creative Commons Attribution (CC BY) license (http://creativecommons.org/licenses/by/4.0/).
